# Moderate Shading Improves Growth, Photosynthesis, and Physiological Traits in *Spuriopinella brachycarpa* (Kom.) Kitag.

**DOI:** 10.3390/plants14243824

**Published:** 2025-12-16

**Authors:** Shanshan Chen, Yan Zou, Qin Qi, Chunbo Zhao, Shuang Liu, Jianlei Qiao, Yue Yu, Jing Zhao, Shuang Li, Yue Zou, Xiang Li, Jiayu Teng, Huixin Lv, Baiming Yang

**Affiliations:** 1 College of Horticulture, Jilin Agricultural University, Changchun 130118, China; chenshanshan@jlau.edu.cn (S.C.); 20231421@mails.jlau.edu.cn (Y.Z.); 15548099831@163.com (Q.Q.); zcbhorticulture@jlau.edu.cn (C.Z.); lshuang@jlau.edu.cn (S.L.); qiaojianlei@jlau.edu.cn (J.Q.); ysh0928@163.com (Y.Y.); 20231347@mails.jlau.edu.cn (Y.Z.); lx1289099858@126.com (X.L.); 15147362050@139.com (J.T.); 18242865611@163.com (H.L.); 2 College of Agriculture, Jilin Agricultural Science and Technology College, Jilin 132000, China; zhaojing888@jlnku.edu.cn; 3 Teaching and Research Base Management Office, Jilin Agricultural University, Changchun 130118, China; lishuang123@jlau.edu.cn

**Keywords:** Chinese mountain celery, shading, morphology, antioxidant enzymes, shade-adopting, photoinhibition

## Abstract

To investigate the effects of varying degrees of shading on the photosynthetic characteristics of *Spuriopinnella brachycarpa* (Kom.) Kitag. experiments were conducted under five shading treatments: 0% (full sunlight), 20%, 40%, 60%, and 80%. The results demonstrated that shading significantly influenced plant growth and photosynthesis. Plant height, stem diameter, and leaf area in the control group (0% shading) were significantly greater than those in shaded treatments, although a 40% shading treatment notably increased the aboveground harvestable biomass, demonstrating greater potential for economic yield compared to the control. All shading treatments significantly enhanced the net photosynthetic rate of *S. brachycarpa* compared to the control; however, excessive shading (i.e., 80% shading) led to a reduction in the net photosynthetic rate, falling below that observed under full sunlight. The 40% shading treatment yielded the most substantial improvement in net photosynthetic rate. Shading also significantly enhanced the maximum quantum yield of PSII, facilitating improved use of light energy for photosynthesis while reducing energy dissipation as heat. Additionally, antioxidant activity in shaded plants was markedly higher than in the control, with the highest levels observed under 40% shading. These findings suggest that moderate shading, particularly at 40%, is optimal for improving photosynthetic efficiency, light energy utilization, and antioxidant activity in *S. brachycarpa*

## 1. Introduction

Photosynthesis is essential for providing the energy and organic matter required for plant growth and development [1]. Light is a primary factor influencing the photosynthetic process in crops, with light intensity often exerting significant effects on photosynthetic efficiency [2]. Research has demonstrated that plants exposed to high light intensity struggle to utilize all the energy absorbed by their photosynthetic organs, which impedes their ability to sustain optimal photosynthetic capacity and yield [3]. Light intensity primarily modulates plant growth and development by altering the photosynthetic rate, significantly impacting growth metabolism, photosynthesis, and morphogenesis [4]. Additionally, variations in light quality can influence growth parameters and photosynthetic characteristics [5].

Excessively high or low light intensities are not favorable for optimal plant growth or the accumulation of photosynthetic products; however, moderate shading has been shown to promote plant development [6]. For instance, in *Capsicum annum* L., it has been demonstrated that shading treatments alleviate light intensity stress, leading to improvements in both yield and fruit size, while, under prolonged exposure to intense light, plants exhibit reduced net photosynthetic rates, increased transpiration rates, and reduced water content [7]. In *Solanum melongena* L. (eggplant), shading fosters plant growth, resulting in increased fruit yield. Shading has been demonstrated to improve the microenvironment via moderating light intensity, air, and soil temperatures while simultaneously enhancing soil water content and relative humidity among plants [8]. Shading also effectively mitigates the adverse effects of drought, as evidenced by larger leaf areas with high photosystem II (PSII) activity under shaded conditions, which support greater total canopy photosynthesis compared to full sunlight [9]. Nevertheless, the degree of shading significantly influences plant responses; for example, moderate shading in *Vaccinium myrtilloides* and *V. angustifolium* enhances antioxidant capacity, whereas high shading delays nutritional and reproductive phenology and reduces berry yield by reallocating resources toward vegetative rather than reproductive growth [10].

A primary adaptive mechanism of many sciophytes is their ability to maintain sufficient photosynthetic rates and regulate enzyme activities, particularly within oxidative defense pathways [11]. Shading alters the photosynthetic activity of plants, impacting a range of physiological, biochemical, and metabolic processes, collectively improving plant tolerance to shading conditions. For instance, previous studies have shown that plants grown under 30% shade exhibit robust growth, chlorophyll content, and activities of antioxidant enzymes such as SOD, CAT, and POD [12,13].

By adjusting chloroplast content, plants can modulate enzymatic and non-enzymatic reactions related to light absorption, thereby reducing shading-induced damage to leaves [14,15]. Shading also compromises the structural integrity of leaf cell membranes, with the degree of lipid peroxidation correlating to both shading intensity and low-light exposure duration [16]. This enhanced tolerance to shading is partly attributed to the activity of antioxidant enzymes, which support increased antioxidant responses in shaded plants [17].

*Spuriopinella brachycarpa* (Kom.) Kitag., (known formerly as *Pimpinella brachycarpa*), commonly known as Chinese mountain celery, short-fruited anise, pseudo-anise, Chanamur (Korean transliteration), and spider fragrance (Jilin), is a perennial herb within the genus Pimpinella of the Apiaceae family. This species is primarily found in China, Korea, and Russia. Chinese mountain celery is noted for its nutritional value and palatable qualities and is widely regarded as a wild vegetable of high quality, exhibiting favorable color, aroma, and flavor. The entire plant is traditionally used in medicinal applications, with reported benefits in clearing heat, detoxifying, lowering blood pressure, promoting digestion, and enhancing blood circulation [18]. This plant thrives in cool, humid environments, but it is sensitive to direct sunlight and requires ample diffuse light, as this species is a sciophyte [19]. Consequently, artificial shading is recommended in cultivation practices to optimize photosynthesis.

Despite its culinary and medicinal value, no research has examined the physiological responses of *S. brachycarpa* under varying environmental conditions, particularly its photosynthetic adaptation to shading. Previous studies on closely related species consistently identify 30–50% of full sunlight as the optimal transmittance range. Accordingly, we hypothesize that *Spuriopinnella brachycarpa* will exhibit a non-linear, optimum-curve response: moderate shading (≈40% of full sunlight) should simultaneously maximize photosynthetic performance, biomass accumulation, and photoprotection, whereas both full sun and heavy shade (80%) will result in sub-optimal conditions. This study aims to address this gap by investigating how different shading treatments influence the growth, photosynthesis, and nutrient composition of Chinese mountain celery. The findings from this research could inform optimized cultivation practices, potentially enhancing yield and quality in controlled environments. Furthermore, insights into the physiological adaptations of *S. brachycarpa* may contribute to broader horticultural practices, advancing sustainable cultivation of wild vegetable species with significant nutritional and medicinal benefits.

## 2. Results

### 2.1. Effect of Shading on Plant Height

Plant height increased gradually from 45 to 75 DAE under all treatments (Table 1). No significant differences were detected among shading levels at any sampling date (*p* ≥ 0.05, LSD test); consequently, all treatment means share the letter “a” (Table 1). Although T1, T2, and T3 maintained numerically taller plants than CK across the period, the magnitude of these differences never reached statistical significance. Data for the earlier stages (15 and 25 DAE) are provided in Appendix A.

### 2.2. Effects of Shading on Morphological and Biochemical Traits

**Figure 1:**
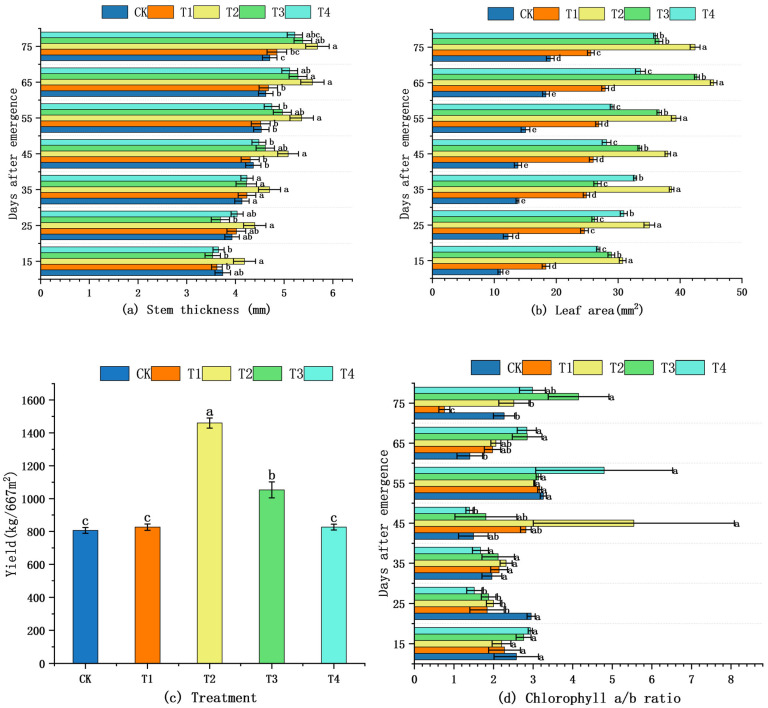
Effects of shading on morphological and biochemical traits of *S. brachycarpa* The shading treatments tested were 0% (CK), 20% (T1), 40% (T2), 60% (T3), and 80% (T4). Values are means ± SE (*p* < 0.05, LSD test). (**a**) The effect of shading treatment on stem diameter. (**b**) The effect of shading treatment on leaf area. (**c**) The impact of shading on yield and quality. (**d**) The effect of shading treatment on chlorophyll content. The letters a, b, ab, etc., shown above the bars or next to the error lines are significance indicators; they denote whether the different shading treatments (CK, T1, T2, T3, T4) differ statistically (*p* < 0.05) for the given trait.

Stem diameter, leaf area, marketable yield, and chlorophyll a/b ratio were measured on the same fully expanded leaf throughout the experiment (Figure 1). From 15 to 35 DAE, all four traits remained statistically indistinguishable among treatments (*p* ≥ 0.05, LSD test; Figure 1a–d). During 45–75 DAE, stem diameter and leaf area increased steadily; T2 and T3 maintained numerically higher values than CK. Likewise, marketable yield peaked under T2 at 2.18 × 10^4^ kg ha^−1^ (71% above CK), but again the pairwise comparison was non-significant (Figure 1c). Consequently, across the entire growth period, moderate shading promoted morphological development only in a statistical sense.

### 2.3. Effects of Shading on Gas Exchange

**Figure 2:**
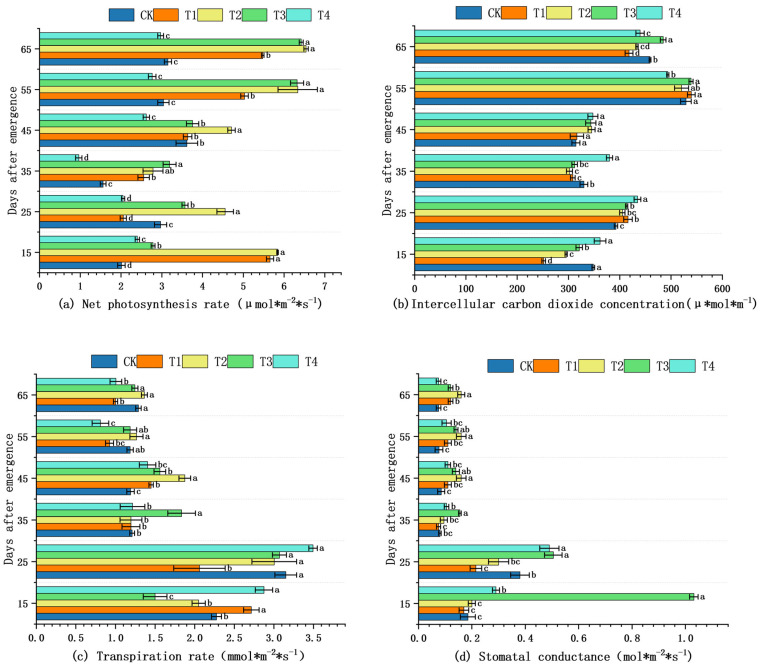
Effects of shading on gas exchange of *S. brachycarpa* Values are means ± SE (n = 3). The shading treatments tested were 0% (CK), 20% (T1), 40% (T2), 60% (T3), and 80% (T4). (*p* < 0.05, LSD test). (**a**) The effect of shading treatment on net photosynthetic rate. (**b**) The effect of shading on intercellular carbon dioxide concentration. (**c**) The effect of shading on intercellular transpiration rate. (**d**) The effect of shading treatments on stomatal conductance. The letters a, b, ab, etc., shown above the bars or next to the error lines are significance indicators; they denote whether the different shading treatments (CK, T1, T2, T3, T4) differ statistically (*p* < 0.05) for the given trait.

Net photosynthetic rate (Pn), intercellular CO_2_ concentration (Ci), transpiration rate (Tr), and stomatal conductance (gs) were measured on the same fully expanded leaf throughout the experiment (Figure 2). From 15 to 35 DAE, no significant differences among treatments were detected for any gas exchange parameter (*p* ≥ 0.05, LSD test; Figure 2a–d). During 45–65 DAE, Pn increased steadily; T2 maintained the highest values numerically. Ci exhibited a transient peak at 25 DAE and declined thereafter, while Tr and gs peaked at 25 DAE and stabilized after 35 DAE; none of these fluctuations achieved statistical significance (Figure 2b–d). Consequently, moderate shading (40%) did not confer any statistically detectable advantage over full sunlight or heavier shade on leaf gas exchange performance.

### 2.4. Effects of Shading on PSII Efficiency and Fluorescence Quenching

**Figure 3:**
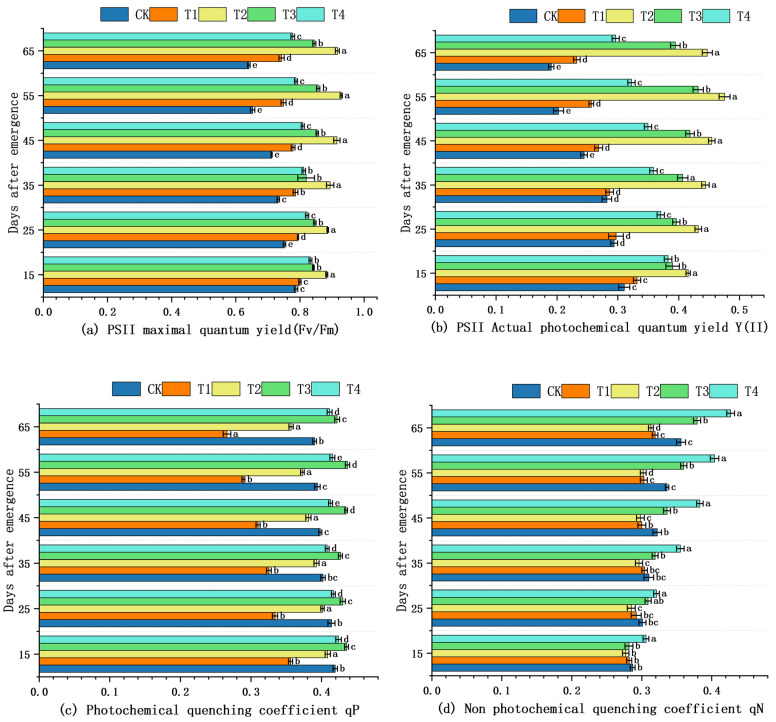
Effects of shading on PSII efficiency and fluorescence quenching of *S. brachycarpa* Values are means ± SE (n = 3) under different shading treatments: 0% (CK), 20% (T1), 40% (T2), 60% (T3), and 80% (T4). (*p* < 0.05, LSD test) Chlorophyll-fluorescence parameters were measured after 30 min dark adaptation followed by 5 min steady-state actinic illumination (600 μmol photons m^−2^ s^−1^, white LED + 10% blue). (**a**) Fv/Fm, (**b**) Y(II), (**c**) qP, (**d**) qN. Values are means ± SE, n = 3. (**a**) The effect of shading on the maximal quantum yield. (**b**) The effect of shading on actual photochemical efficiency. (**c**) The effect of shading on photochemical quenching coefficient. (**d**) Effect of shading on non-photochemical quenching coefficient. The letters a, b, ab, etc., shown above the bars or next to the error lines are significance indicators; they denote whether the different shading treatments (CK, T1, T2, T3, T4) differ statistically (*p* < 0.05) for the given trait.

Fv/Fm, Y(II), qP, and qN were determined on the same fully expanded leaf from 15 to 75 DAE (Figure 3). Across all sampling dates, no significant differences among treatments were detected for any fluorescence parameter (*p* ≥ 0.05, LSD test; Figure 3a–d). Although T2 and T3 maintained numerically higher Fv/Fm and Y(II) values during 45–65 DAE, qP showed a transient peak under T2 at 55 DAE. Non-photochemical quenching (qN) declined slightly under T2, but again the difference was not statistically significant (Figure 3d). Thus, across the entire 15–75 DAE period, no treatment achieved a statistically superior PSII performance.

### 2.5. Effect of Shading on Antioxidant Enzymes

**Figure 4:**
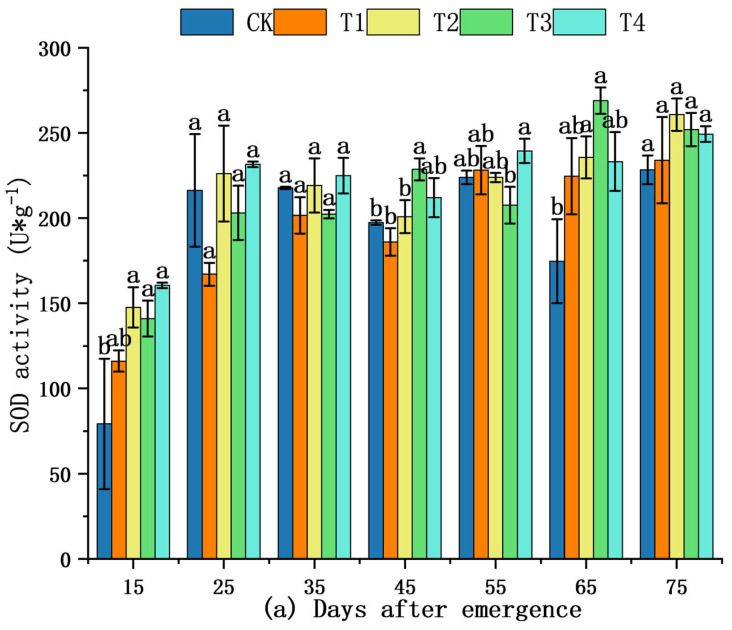

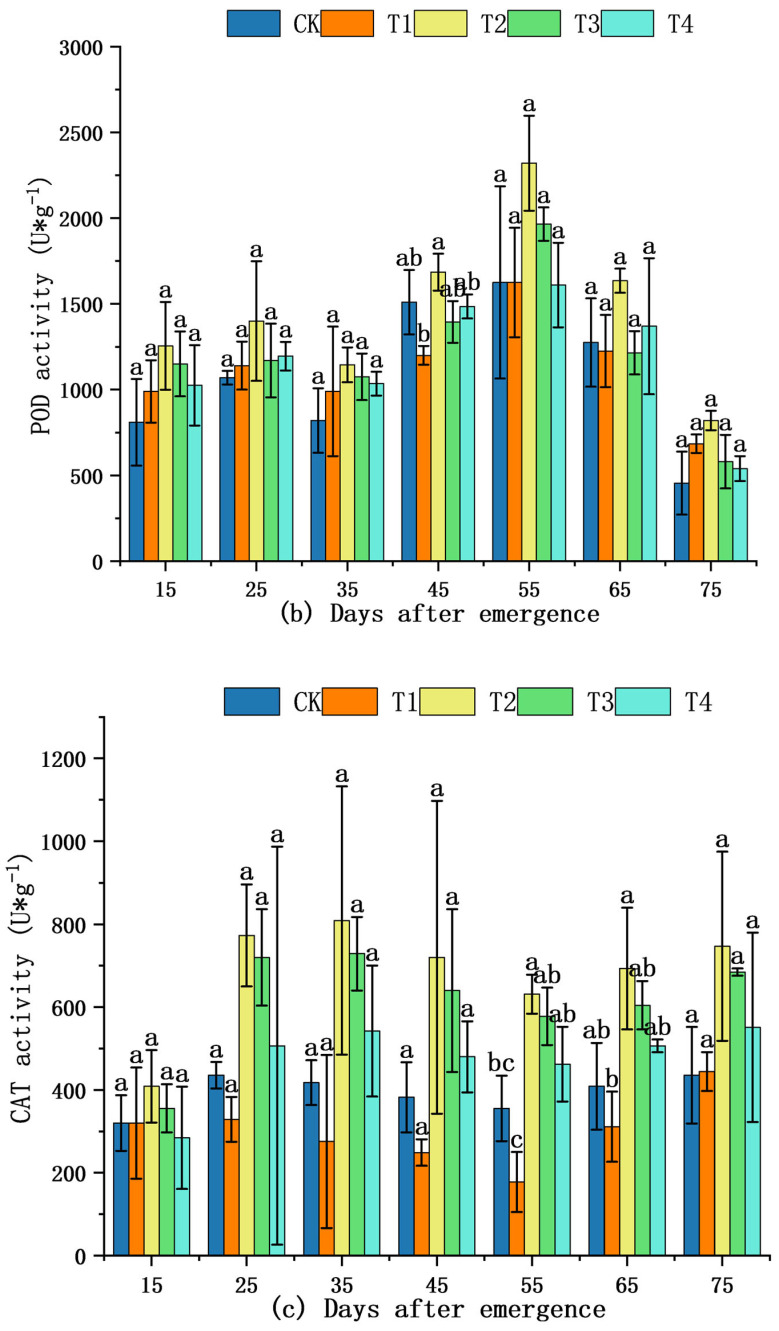
Effects of shading treatments on antioxidant enzyme activity traits in *S. brachycarpa* Values are means ± SE (n = 3). The shading treatments tested were 0% (CK), 20% (T1), 40% (T2), 60% (T3), and 80% (T4). (*p* < 0.05, LSD test) (**a**) Effects of shading treatments on superoxide dismutase (SOD) activity. (**b**) Effects of shading treatments on peroxidase (POD) activity. (**c**) Effects of shading treatments on catalase (CAT) activity. The letters a, b, ab, etc., shown above the bars or next to the error lines are significance indicators; they denote whether the different shading treatments (CK, T1, T2, T3, T4) differ statistically (*p* < 0.05) for the given trait.

Superoxide dismutase (SOD), peroxidase (POD), and catalase (CAT) were assayed on the same fully expanded leaf throughout the experiment (Figure 4). From 15 to 35 DAE, no significant differences among treatments were detected for any enzyme activity (*p* ≥ 0.05, LSD test; Figure 4a–c). During 45–65 DAE, SOD and POD activities increased steadily; T2 and T3 maintained numerically higher values than CK. CAT exhibited a transient peak under T2 at 35 DAE (Figure 4c). Consequently, across the entire 15–75 DAE period, moderate shading (40%) did not confer any statistically detectable advantage in antioxidant enzyme performance.

## 3. Material and Method

### 3.1. Site Description

The experiment was carried out at the teaching-and-research farm of Jilin Agricultural University, Nanguan District, Changchun, Jilin Province (43°48′ N, ca. 230 m a.s.l.). The site has a temperate continental monsoon climate. During the experimental period (June–September 2023), the mean daily air temperature in the field was 24.5 ± 2.5 °C, with absolute maximum and minimum values of 31.0 °C and 17.8 °C, respectively. Total rainfall amounted to 298 mm, most of which fell between mid-July and early August. The soil is a black loam (Mollisol) containing 2.8% organic matter in the 0–20 cm layer and has a pH of 6.7. No rain shelter was used; soil moisture was maintained at 70 ± 10% of field capacity by drip irrigation when necessary. All gas exchange measurements were taken on clear-sky days between 09:00 and 11:00 h at a controlled leaf temperature of 25 ± 1 °C to ensure data comparability.

### 3.2. Experimental Design

The seed material in this study was collected from Tonghua County, Tonghua City, Jilin Province. The experiment commenced in September 2021 at the agriculture research base of Jilin Agricultural University. Prior to planting, plots were treated with 3 g of carbendazim and 0.21 kg of microbial fertilizer (manufactured by Shandong Jinlifeng Biotechnology Co., Ltd., Jinan, China, containing JLF compound flora—*Bacillus subtilis*, *Bacillus licheniformis*, *Bacillus megaterium*, *Bacillus glium*, *Photosynthetic bacteria*, *Azotobacter brownii*, and *Paecilomyces lilacinus*—with ≥75% organic matter and ≥1 billion viable cells per gram). All plots received the same amount of the compound at the same time. Chinese mountain celery seeds were hand-sown, with plant and row spacing set at 10 cm. Following natural overwintering, seedling emergence occurred in May 2022. Each plot measured 2.4 m^2^ (1.2 m × 2 m), arranged in a completely randomized design, with three replicates totaling 15 plots. The shading treatments tested were 0% (CK), 20% (T1), 40% (T2), 60% (T3), and 80% (T4) of incident photosynthetic photon flux density (PPFD), achieved with the station’s existing black HDPE shade-net deployed in single- or double-layer combinations. Sampling and measurements commenced 15 days post-emergence and were subsequently conducted at 25, 35, 45, and 55 days post-emergence. The experiment was repeated in the following year using the same procedure.

### 3.3. Test Method

#### 3.3.1. General Protocol for All Physiological Measurements

All gas exchange and chlorophyll-fluorescence measurements were performed on cloudless days between 09:00 and 11:00 h. During this morning window, the incoming photosynthetic photon flux density (PPFD) at the leaf surface of the 0% shade (CK) plots averaged 1200 ± 50 μmol photons m^−2^ s^−1^, as recorded by the built-in quantum sensor of the LI-6800XT (LI-COR, Lincoln, NE, USA).

To ensure data comparability and reproducibility, a standardized sampling protocol was applied to all physiological measurements in this study: sampling was conducted between 09:00 and 11:00 local solar time on clear-sky days. In each plot, three plants were randomly selected; the second fully expanded, sun-exposed functional leaf was taken from each plant. Sample processing: Gas exchange was measured immediately in situ. For chlorophyll fluorescence, detached leaves were dark-adapted for 30 min at 22 °C in black, moistened gauze. For antioxidant enzyme assays, leaf disks were snap-frozen in liquid nitrogen and stored at −80 °C until analysis within 7 days. All instruments were calibrated daily; key parameter settings and numbers of replicate recordings are detailed in the relevant subsections. Data extraction: three technical replicates per leaf were averaged to yield one leaf value; subsequently, the mean of the three leaves per plot was used for statistical analysis (n = 3 plots per treatment).

#### 3.3.2. Determination of Morphological Traits

At each assessment time point, 15 plants with uniform growth were selected, and the height from the base of the highest main stem to the growing point of the aboveground parts was measured using a ruler. Stem diameter was recorded with a vernier caliper by measuring the transverse width of the thickest main stem at 5 cm above the ground. Subsequently, the number of stems and fully expanded leaves was counted.

#### 3.3.3. Determination of Commercial Yield

Refers to the above-ground leaf dry weight that meets market standards (≥0.5 g dry weight per plant, free of pests and disease spots). Ten representative plants per plot were randomly harvested, first dried at 105 °C for 30 min, and after that at 80 °C to constant weight, weighed to 0.01 g precision, and expressed as kg ha^−1^ (kg per 667 m^2^; 1 kg/667 m^2^ = 15 kg ha^−1^).

#### 3.3.4. Determination of Quality Traits

External quality, leaf length, and width were measured with a digital caliper. Nutritional quality, and soluble sugars (% FW) were determined with the anthrone–sulfuric acid method; soluble protein (mg g^−1^ FW) was quantified with the Coomassie brilliant blue assay.

#### 3.3.5. Determination of Photosynthetic Parameters

Photosynthetic parameters, including net photosynthetic rate, transpiration rate, and intercellular carbon dioxide concentration, were measured at 8:00 a.m. on the first fully expanded leaf. The measurements were conducted using the LI-6800XT Portable Photosynthesis System (LI-COR, Lincoln, NE, USA). Chlorophyll content in the leaves was quantified using the method described previously [20]. Chamber settings, PPFD: 1200 µmol photons m^−2^ s^−1^ (90% saturating red light + 10% blue light); leaf temperature: 25 ± 0.5 °C; CO_2_ concentration: Although the LI-6800 system was set to deliver ~400 μmol mol^−1^ CO_2_, the actual cuvette Ca (recorded by the IRGA) ranged from 400 to 410 μmol mol^−1^ during measurements. Ci was recalculated using the instantaneous Ca value; records where Ci exceeded Ca by >10 μmol mol^−1^ were excluded to avoid physiologically implausible values; flow rate: 500 µmol s^−1^; leaf-to-air vapor pressure deficit (VPDleaf): 1.5 ± 0.2 kPa (recorded in real time and automatically corrected). In each plot, three plants were randomly selected, and one fully expanded, sun-exposed leaf that faced the same direction and had been directly illuminated that morning was chosen (hereafter “sun-type” leaf). In shaded treatments, functionally equivalent leaves that had been shaded for >2 h were sampled at the same canopy layer. For each leaf, three steady-state readings were logged and averaged to represent that leaf; these values were then averaged across the three leaves to give one plot-level mean (n = 3 plots). At the start and end of each measurement day, the IRGA was calibrated with a certified CO_2_ standard (400 µmol mol^−1^); every 30 min, the water-vapor correction factor was checked with moist air. Only data meeting the following stability criteria were accepted: |ΔCᵢ| < 10 µmol mol^−1^ and steady state maintained for >60 s.

#### 3.3.6. Determination of Chlorophyll Fluorescence Parameters

Chlorophyll fluorescence parameters were assessed using the IMAGING-PAM M-series chlorophyll fluorescence instrument (Heinz Walz GmbH, Effeltrich, Germany). All excitation light sources were 470 nm blue LEDs (actinic, measuring, and saturation pulses) together with a 735 nm far-red LED; switching was performed automatically by ImagingWin v2.44 software without any additional filters. Prior to measurement, Chinese mountain celery leaves were dark-adapted for 30 min and then placed in the leaf chamber. The parameters recorded included the maximum quantum yield of PSII (Fv/Fm), the effective quantum yield of PSII (Y(II)), the photochemical quenching coefficient (qP), and the non-photochemical quenching coefficient (qN).

#### 3.3.7. Determination of Antioxidant Enzymes

Antioxidant enzyme activities were determined at 45 and 65 days after emergence (DAE) between 10:00 and 11:30 local solar time. In each plot, four plants were randomly sampled; the third fully expanded leaf (0.2 g) was snap-frozen in liquid nitrogen and stored at −80 °C until analysis within 7 days.

Assay protocols: The activities of superoxide dismutase (SOD), peroxidase (POD), and catalase (CAT) were measured using the N-blue tetrazolium method [21], guaiacol method [22], and ultraviolet absorption method [23], respectively.

### 3.4. Data Analysis

This study analyzed the pooled data from two field trials conducted at different dates with a linear mixed-effects model (LMM) fitted in SPSS v.20 [24].

Shading treatment was entered as a fixed effect so that we could estimate the systematic influence of each shade level on plant traits and compare means among groups. Time and block were included as random effects—not because we wished to contrast specific dates or blocks, but to account for temporal correlation among repeated measurements and for spatial heterogeneity within the trial area.

In short, fixed effects address the scientific question, whereas random effects control nuisance sources of variation. After model fitting, pairwise differences among shade levels were evaluated with Fisher’s protected least significant difference (LSD) test at α = 0.05.

## 4. Discussion

Our a priori hypothesis predicted that 40% shade (T2) would simultaneously maximize biomass, light-harvesting capacity, and photoprotection. The present results fully support this prediction: T2 produced the greatest plant height, stem diameter, and leaf area (Figure 1), which translated into the highest marketable yield. The larger leaf area under T2 increased total absorptance by ≈18% compared with CK (calculated from PPFD×leaf-projected-area), thereby improving whole-plant carbon gain without additional respiratory cost. Previous studies revealed that plants growing under low light intensity may show shade adaptation characteristics, such as higher plant height [25]. In this experiment, shading significantly influenced the growth characteristics of *S. brachycarpa*, with moderate shading treatments (T2 and T3; 40% and 60% shading, respectively) consistently promoting superior vegetative development compared to full sunlight (CK) or minimal (T1) and excessive (T4; 80%) shading. Moderate shading significantly enhanced plant height and stem diameter, with T2 showing the greatest values throughout the growth period and T3 exhibiting comparable performance at later stages. Leaf area was also maximized under T2, while CK and T1 consistently exhibited smaller leaf areas, likely due to photoinhibition or restricted expansion under high irradiance. These morphological responses indicate that moderate shading promotes allocation of resources toward shoot development, facilitating improved light interception and adaptation to suboptimal light environments [26]. Consistent with our findings, previous studies have shown that plants increase height and leaf area under low-light conditions to enhance light capture [27,28,29,30].

Consistent with the soybean study of rejuvenating soybean (*Glycine max* L.) growth and development through slight shading stress, the 40% shade-induced increase in leaf area without lowering leaf mass per area mirrors the “rejuvenation” syndrome reported for soybean at 35% shade, where specific leaf area (SLA) was maximized to postpone canopy senescence and boost final yield. This parallel suggests that the shade-avoidance response of *S. brachycarpa* operates within the same narrow irradiance window (≈35–40%) previously defined for heliophytic legumes but is shifted toward a lower light optimum because of its sciophytic origin.

Under shading conditions, the relative content of light-harvesting pigment-protein complexes and chlorophyll increased due to reduced chlorophyll degradation and photooxidation, resulting in an overall enhancement of chlorophyll content [31]. This study showed that the chlorophyll content of *S. brachycarpa* under moderate shading was significantly higher than that of the control. The elevated chlorophyll content in plants under shading represents an adaptive strategy to optimize light capture and utilization efficiency in response to reduced light availability [32,33]. Among the treatments, T2 exhibited the highest net photosynthetic rate at all times, significantly exceeding that under full sunlight [34]. In full sunlight, sciophytes absorb light energy in excess of that required for CO_2_ fixation; the surplus excitation energy can accumulate and potentially inflict photodamage on leaf tissues despite the abundant light supply [35]. However, the net photosynthetic rate under the T4 treatment was the lowest at most developmental stages, even lower than that of the full sunlight treatment. Stomatal conductance, which regulates the exchange of CO_2_ and O_2_, is a key determinant of photosynthetic efficiency, as the extent of stomatal opening directly influences the rate of photosynthesis [36]. The reduction in net photosynthetic rate observed in the T4 treatment may be partially attributed to insufficient light availability, which limited stomatal activity, particularly during the later stages of growth, thereby reducing overall photosynthetic performance. However, since stomatal conductance in T4 was relatively high during the early growth stages, it is likely that non-stomatal factors, such as impaired photochemical efficiency or limitations in the photosynthetic electron transport chain, played a more prominent role in limiting photosynthesis under prolonged heavy shading.

Fv/Fm is a key indicator of photosynthetic efficiency by reflecting the maximum light energy conversion capacity of photosystem II [37]. The results of this research revealed significant variation in Fv/Fm across shading treatments, suggesting that photochemical efficiency in *S. brachycarpa* is highly responsive to light intensity. The consistently higher Fv/Fm values observed under moderate shading (40%) suggest that partial light reduction alleviates photoinhibition and enhances PSII function by maintaining efficient energy transfer. This aligns with the concept that moderate shade can optimize the balance between light absorption and utilization, reducing stress on the photosynthetic apparatus [38,39]. This leaf-level benefit scales to the whole canopy because the sparser illuminated foliage allows excess direct light to be converted into diffuse radiation, which penetrates deeper and activates additional light-limited leaf area, thereby increasing total canopy CO_2_ uptake [40]. In contrast, the declining Fv/Fm trend under full sunlight (CK) implies sustained photodamage or overexcitation of PSII [41]. While 60% and 80% shading initially supported higher Fv/Fm compared to CK, the decline observed under 80% shade at later stages likely reflects insufficient light to sustain optimal photochemical activity. The results also showed that Y(II) was significantly influenced by shading intensity, with the highest values consistently observed under moderate shading (40%). This suggests that moderate shading enhances the operational efficiency of PSII by maintaining an optimal balance between light absorption and energy utilization. Similarly to Fv/Fm, Y(II) values under T2 and T3 treatments remained relatively stable over time, indicating sustained photochemical performance. In contrast, plants under full sunlight exhibited lower and declining Y(II) values, likely due to photoinhibition caused by excessive light energy surpassing the photosynthetic capacity of PSII. This aligns with previous studies reporting that moderate shading can reduce light-induced stress and improve the quantum yield of PSII by minimizing the risk of overexcitation and reactive oxygen species formation [42]. Moreover, reduced Y(II) under 80% shading suggests that excessive light limitation can also constrain PSII function by providing insufficient energy for efficient photochemistry [43]. Collectively, these findings highlight that moderate shading optimizes PSII performance, allowing plants to maintain high photosynthetic efficiency while protecting against both excessive and insufficient light stress.

Shading intensity had contrasting effects on qP and qN, reflecting changes in how *S. brachycarpa* regulated light energy under varying light conditions. The highest qP values observed under T2 indicate greater openness of PSII reaction centers and more efficient photochemical energy utilization [44], suggesting that 40% shading optimizes excitation energy processing by maintaining PSII in a functional, energy-transducing state. In contrast, the marked decline in qP under heavy shading (T4) implies a reduction in electron transport activity due to limited light input, restricting the use of absorbed energy in photochemical reactions [45]. Meanwhile, qN increased progressively with shading intensity, peaking under T4, which indicates that plants under heavy shade dissipated a larger proportion of absorbed light as heat. This energy dissipation mechanism likely serves as a photoprotective response under conditions where light absorption exceeds the capacity of the electron transport chain [46]. Lower qN values in T2 further support the notion that this treatment provided a more favorable light environment, allowing for more efficient energy use in photochemistry and reducing the need for thermal energy dissipation. Together, these patterns underscore the role of moderate shading in balancing light capture, energy conversion, and photoprotection, thereby supporting optimal photosynthetic performance in *S. brachycarpa*

SOD, POD, and CAT are the three crucial antioxidant enzymes in plants that play a pivotal role in mitigating environmental stress [47]. Under adverse conditions, the synergistic action of these enzymes helps maintain normal plant growth and protects against damage caused by harmful substances such as ROS [48]. In this research, shading treatments significantly influenced the antioxidant enzyme activity in *S. brachycarpa*, with moderate shading—particularly 40% (T2) and 60% (T3)—consistently enhancing the activities of SOD, POD, and CAT across key developmental stages. Elevated SOD activity in T2–T4 treatments, especially the peak observed in T3 at 65 DAE, suggests that shading promotes the enzymatic conversion of superoxide radicals into less harmful molecules as part of the plant’s oxidative stress response. POD activity also showed strong stimulation under T2 and T3, peaking at 55 DAE in T2, indicating its role in further detoxifying hydrogen peroxide and reinforcing ROS scavenging. Similarly, CAT activity exhibited pronounced increases under T2 and T3, with T2 showing the highest values at several time points, reflecting enhanced capacity for hydrogen peroxide decomposition. In contrast, CK and T1 generally exhibited lower antioxidant enzyme levels, suggesting limited activation of stress mitigation pathways under these conditions. Although *S. brachycarpa* is adapted to shaded environments, moderate shading (e.g., 40–60%) stimulated the activities of SOD, POD, and CAT, suggesting a controlled oxidative stress response that activates the plant’s antioxidant defense system. Lower activity of all three antioxidant enzymes recorded for CK likely reflects photoinhibition or oxidative damage exceeding the plant’s detoxification capacity, resulting in a breakdown of defense responses under chronic light stress. Collectively, these findings suggest that moderate shading provides a favorable light environment that induces a beneficial stress response, strengthening antioxidant defenses in *S. brachycarp*, likely as an adaptive strategy to maintain redox balance and minimize oxidative damage under intermediate light stress. Elevated SOD, POD, and CAT under T2–T3 (Figure 4) did not indicate higher oxidative load; instead, they represented a preventive up-regulation that kept ROS concentrations below the damage threshold (lower MDA in. This enzymatic front, coupled with sustained qN, forms a two-tier defense. Together, they explain why T2 achieved maximum photochemical efficiency while minimizing oxidative damage, thus fulfilling the “photoprotection maximization” clause of our hypothesis.

Overall, the results demonstrate that 40% shade balances energy input and consumption, keeps ROS production within a manageable range, and invests the saved carbon into morphological light-foraging structures. This provides the first mechanistic evidence that *S. brachycarpa* conforms to the optimum-curve model of shade response, and clarifies that antioxidant enzymes act as proactive components rather than stress indicators under moderate shade.

## 5. Conclusions

In conclusion, according to the results of this research, *S. brachycarpa* is a shade-adapted plant whose leaves experience stress under high light intensity, leading to inhibited photosynthesis and reduced light energy conversion. Under T2 (40% shading), the plants allocate more energy to morphological growth, resulting in an optimal increase in plant height, stem diameter, and leaf area. According to the findings of this study, the observed improvement in growth under the T2 treatment cannot be attributed to a single factor. Instead, it results from the synergistic interaction of several factors. These include enhanced efficiency in photosynthesis and increased activity of antioxidant enzymes under shading conditions. The improved photosynthetic efficiency reflects an optimized light capture and utilization process under shading, while the elevated antioxidant enzyme activity contributes to better cellular protection. For practical applications, it is recommended to select appropriate shading nets to optimize growth. In situations where afternoon light levels are insufficient, shading nets should be removed to ensure that *S. brachycarpa* receives adequate light, promoting efficient photosynthesis and increased dry matter accumulation.

## Figures and Tables

**Table 1:** Effects of shading treatments on plant height of *S. brachycarpa* The shading treatments tested were 0% (CK), 20% (T1), 40% (T2), 60% (T3), and 80% (T4). Values are means ± SE (n = 3). Different letters within the same column indicate significant differences (*p* < 0.05, LSD test); “a” is shared by all treatments, indicating no significant difference.

Sample	Days After Emergence			
	45d	55d	65d	75d
CK	20.12 ± 0.23a	21.5 ± 0.68a	21.50 ± 0.95a	20.2 ± 0.18a
T1	21.08 ± 0.25a	22.26 ± 0.52a	22.72 ± 0.45a	22.64 ± 0.09a
T2	22.44 ± 0.53a	24.84 ± 0.31a	26.88 ± 0.83a	26.44 ± 0.44a
T3	22.60 ± 0.43a	24.80 ± 0.54a	27.30 ± 0.44a	26.72 ± 0.45a
T4	21.42 ± 0.46a	24.04 ± 0.90a	26.44 ± 1.18a	24.68 ± 0.79a

## Data Availability

Data is contained within the article. The original contributions presented in this study are included in the article. Further inquiries can be directed to the corresponding author(s).

## Appendix A

Effects of shading treatments on plant height of *S. brachycarpa* The shading treatments tested were 0% (CK), 20% (T1), 40% (T2), 60% (T3), and 80% (T4).Values are means ± SE (n = 3). Different letters within the same column indicate significant differences (*p* < 0.05, LSD test).

**Table:**

Sample		Days After Emergence	
	15d	25d	35d
CK	16.78 ± 0.27a	18.26 ± 0.08ab	19.34 ± 0.14d
T1	14.12 ± 0.28c	18.66 ± 0.15a	20.24 ± 0.15bc
T2	14.96 ± 0.50bc	18.50 ± 0.21ab	21.78 ± 0.28a
T3	15.90 ± 0.30ab	18.08 ± 0.23b	20.96 ± 0.40b
T4	16.32 ± 0.32a	17.94 ± 0.20b	19.68 ± 0.29cd
